# Sequencing for germline mutations in Swedish breast cancer families reveals novel breast cancer risk genes

**DOI:** 10.1038/s41598-021-94316-z

**Published:** 2021-07-19

**Authors:** Hafdis T. Helgadottir, Jessada Thutkawkorapin, Kristina Lagerstedt-Robinson, Annika Lindblom

**Affiliations:** 1 Department of Molecular Medicine and Surgery, Karolinska Institute, Solna, Stockholm, Sweden; 2 Department of Clinical Genetics, Karolinska University Hospital, Solna, Stockholm, Sweden

**Keywords:** Breast cancer, Cancer, Genetics, Cancer

## Abstract

Identifying genetic cancer risk factors will lead to improved genetic counseling, cancer prevention and cancer care. Analyzing families with a strong history of breast cancer (BC) has been a successful method to identify genes that contribute to the disease. This has led to discoveries of high-risk genes like the BRCA-genes. Nevertheless, many BC incidences are of unknown causes. In this study, exome sequencing on 59 BC patients from 24 Swedish families with a strong history of BC was performed to identify variants in known and novel BC predisposing genes. First, we screened known BC genes and identified two pathogenic variants in the *BRIP1* and *PALB2* genes. Secondly, to identify novel BC genes, rare and high impact variants and segregating in families were analyzed to identify 544 variants in novel BC candidate genes. Of those, 22 variants were defined as high-risk variants. Several interesting genes, either previously linked with BC or in pathways that when flawed could contribute to BC, were among the detected genes. The strongest candidates identified are the *FANCM* gene, involved in DNA double-strand break repair, and the *RAD54L* gene, involved in DNA recombination. Our study shows identifying pathogenic variants is challenging despite a strong family history of BC. Several interesting candidates were observed here that need to be further studied.

## Introduction

Breast cancer (BC) is the most common cancer in women and the leading cause of cancer death^1^. Hereditary risk factors account for many incidences, where having a close relative with BC increases the risk substantially. It is estimated that about 20–30% of all new BC cases are due to hereditary risk factors^2,3^ and high-risk genes account for 5–10%^2^.

Analyzing families to identify variants shared by affected individuals has resulted in the identification of the *BRCA1* and *BRCA2* genes^4–6^. The *BRCA1/2* genes are the most common high-risk genes accounting for 15% of the familial cases and result in up to 60–85% risk of developing BC^6^. Additional high-risk genes have been identified by the candidate gene approach where genes with a function that could contribute to BC, such as DNA repair mechanism, have been screened resulting in the identification of *CHEK2*, *ATM*, *PALB2,* and *BRIP1*^7^.

Despite the identification of strong genetic risk factors, many BC incidences are of unknown genetic causes. With improved technology and increased sample collection, extensive genome-wide association studies (GWAS) have linked more than 170 genomic loci to increased risk of BC^8–13^. However, these loci are common and confer low risk to BC.

Here, we performed exome sequencing on 59 BC patients from 24 Swedish families with the aim of identifying variants that could contribute to BC. First, pathogenic variants in known BC susceptible genes were analyzed. Secondly, rare and high impact variants in new BC candidate genes shared by all affected family members were identified.

## Material and methods

### Families

The individuals in this study were BC patients from families that had undergone genetic counseling at the Department of Clinical Genetics, Karolinska University Hospital Solna, Sweden. All families comprised of at least three close relatives with BC (range 3–8 BC patients). As a part of the study, additional family members were recruited when possible. For each family, one to four individuals were whole-exome sequenced, resulting in 59 BC patients from 24 families represented by of 1st to 4th degree relatives. In total, the study used three families with four sequenced individuals (WES-4s, average age of onset 50.8 ± 6.7 years, consisting of 1st to 3rd degree relatives), six families with three sequenced individuals (WES-3s, average age of onset 49.4 ± 11.9 years, consisting of 1st to 4th degree relatives), 14 families with two sequenced individuals (WES-2s, average age of onset 49.4 ± 10.9 years, consisting of 1st to 4th degree relatives) and one family with one sequenced individual (WES-1s, age of onset 47 years).

All patients gave written informed consent to participate in the study and to donate blood samples. The study was approved by the regional ethics committee in Stockholm. All methods were conducted in accordance with the Declaration of Helsinki guidelines.

### Exome sequencing of BC patients

DNA was quantified using a Qubit Fluorometer (Life Technologies, US). Sequencing libraries were prepared according to the TruSeq DNA Sample Preparation Kit EUC 15005180 or EUC 15026489 (Illumina, US) at an average coverage of 100×. Briefly, 1–1.5 ug of genomic DNA was fragmented (Covaris, Inc., US) and all samples were subjected to end-repair, A-tailing, and adaptor ligation (Illumina Multiplexing PE adaptors). A gel-based size selection step was performed, and the adapter-ligated fragments enriched by PCR, followed by purification using Agencourt AMPure Beads (Beckman Coulter, Sweden). Exome capture was performed by pre-pooling equimolar amounts and performing enrichment in 5- or 6-plex reactions according to the TruSeq Exome Enrichment Kit Protocol (EUC 15013230). Library size was analyzed on a Bioanalyzer High Sensitivity DNA chip (Agilent Technologies, Sweden) and concentration calculated by quantitative PCR. The pooled DNA libraries were clustered on a cBot instrument (Illumina) using the TruSeq PE Cluster Kit v3. Paired-end sequencing was performed for 100 cycles using a HiSeq 2000 instrument (Illumina) with TruSeq SBS Chemistry v3, according to the manufacturer’s protocol. Basecalling was performed with RTA (1.12.4.2 or 1.13.48) and the resulting BCL files were filtered, de-multiplexed, and converted to FASTQ format using CASAVA 1.7 or 1.8 (Illumina).

### Bioinformatics workflow

Sequencing reads were aligned to the reference genome GRCh37 using BWA^14^ and Picard (http://broadinstitute.github.io/picard/) used to mark PCR-duplicated reads. Variants were called using GATK by following the best practice procedure implemented at the Broad Institute^15^. Variant annotation was done by ANNOVAR^16^, including RefSeq gene^17^ and dbSNP150^18^. Max minor allele frequency (MMAF) was calculated from the ExAC^19^, 200Danes^20^, SweGen^21^, and 1000 Genomes Project allele frequencies^22^. To assess and predict pathogenic effects of the variants ClinVar^23,24^, ACMG classification^25^ and the in silico predictor tool CADD^26^ were used. CADD > 20 and CADD > 30 indicate the 1% and 0.1% of most deleterious variants, respectively.

To exclude variants with missing data, BC genotype frequency (BC_GF) was calculated for every variant. A variant with a BC_GF of 0.8 indicates that 80% of the patients had genotypes for that particular variant. No alternative method was used to confirm the genetic variants identified in this study. The presence of high-risk variants was confirmed by manual inspection of the bam files in the IGV software^27^.

### Known BC-predisposing genes: variant selection

Variants in 15 BC and ovarian cancer (OC) genes commonly screened at Karolinska University Hospital as a part of genetic counseling (*ATM, BRCA1, BRCA2, BRIP1, CHEK2, EPCAM, MLH1, MSH2, MSH6, NBN, PALB2, PMS2, RAD51C, RAD51D* and *TP53*) were identified in BC families. All variants that (1) had BC_GF > 0.8; (2) MMAF < 0.2; (3) were not considered benign according to ClinVar; and (4) had CADD > 20 were selected for analysis.

### Novel BC-predisposing genes: variant selection

Variants that were (1) detected in all family members; (2) had BC_GF > 0.8; (3) with MMAF < 0.01; and (4) with CADD > 20 were selected for further analysis. Additionally, variants that were (1) detected in all family members of WES-3s and WES-4s; (2) had BC_GF > 0.8; (3) with MMAF < 0.001; and (4) with CADD > 25 were defined as high-risk variants.

### Ethical statements

All patients gave written informed consent to participate in the study and to donate blood samples. The study was approved by the research ethics committee at Karolinska Institutet and the regional ethics committee in Stockholm. All methods were conducted in accordance with the Declaration of Helsinki guidelines.

## Results

### Pathogenic variants in known BC-predisposing genes were seen in five BC families

Previously, only one affected individual in each family has been tested for variants in known BC and OC-predisposing genes. Therefore, we searched for variants in the 15 genes from the clinical panel (see “Material and methods” section) in all 59 BC patients from the 24 families.

In total, 10 variants were seen in 13 individuals from 9 families (Table 1). Three of the variants were known pathogenic variants: (1) c.2108delTinsGGA (rs786203384, p.(Lys703fs)) in the *BRIP1* gene, (2) c.2748 + 1G > T (rs753153576) in the *PALB2* gene, and (3) c.1100delC (rs555607708) in the *CHEK2* gene. The *BRIP1* frameshift variant and the *PALB2* splice donor variant result in protein truncation and were observed in one family each. *CHEK2* variant c.1100delC was seen in four individuals from three different families, in the WES-2 family Br15 and the WES-3 family Br7 and in two individuals from the WES-4 family Br1 (Table 1). Five additional missense variants listed as VUS (variant of uncertain significance) or conflict interpretation of pathogenicity were detected in the BC families (Table 1).

**Table 1:** Variants in known BC and OC predisposing genes.

Gene	Location Ref > Alt SNPid	Function	Change	ClinVar	MMAF	ACMG classification	CADD	WES	Families	Carriers
*MSH2*	chr2:47703548G > T rs755920849	Missense	NM_000251:exon13 c.G2048T:p.G683V	Uncertain significance	0	3	32	WES-4s	Br2	Br2.2, Br2.4
*NBN*	chr8:90983460G > A rs34767364	Missense	NM_002485:exon6 c.C643T:p.R215W	Conflicting interpretation of pathogenicity	0.006	3	26	WES-1s	Br24	Br24.1
*ATM*	chr11:108188118C > G rs767406075	Missense	NM_000051:exon43 c.C6217G:p.L2073V	Uncertain significance	6.0E−05	3	28.3	WES-2s	Br22	Br22.1, Br22.2
*PALB2*	chr16:23637556C > A rs753153576	Splicing	NM_024675:exon7 c.2748 + 1G > T	Pathogenic/likely pathogenic	1.5E−05	5	26.1	WES-3s	Br4	Br4.1
*RAD51C*	chr17:56787304G > A rs147241704	Missense	NM_058216:exon5 c.G790A:p.G264S	Conflicting interpretations of pathogenicity	0.005	2	23.4	WES-3s	Br5	Br5.2
*BRIP1*	chr17:59821942 T > GGA rs786203384	Insert	NM_032043:exon15 c.2108delTinsGGA:p.Lys703fs	Pathogenic	0	5	na	WES-2s	Br16	Br16.2
*CHEK2*	chr22:29091824G > A rs587780167	Missense	NM_007194:exon11 c.C1133T:p.T378I	Uncertain significance	2. 0E−04	3	23.4	WES-2s	Br16	Br16.2
	chr22:29091857C > - rs555607708	Deletion	NM_007194:exon11 c.1100delC:p.T367fs	Conflicting interpretations of pathogenicity	0.0083	5	na	WES-2s	Br15	Br15.2
								WES-3s	Br7	Br7.3
								WES-4s	Br1	Br1.1, Br1.3

Location according to hg19, SNPid according to dbSNP150, Change shows transcript, exon, amino acid change and protein change. MMAF: Max ref AF indicates the highest reference allele frequency (see methods). WES-4s: families with 4 sequenced individuals; WES-3s: families with 3 sequenced individuals; WES-2s: families with 2 sequenced individuals; WES-1s: families with 1 sequenced individual.

Since family members of families Br4 and Br16 carried clear pathogenic variants in the *PALB2* and the *BRIP1* genes, these two families were excluded from further analysis.

### Nearly 40 pathogenic variants in novel BC candidate genes were seen in BC families

In the remaining 22 BC families we searched for new BC-predisposing genes. All variants that (1) were observed in all family members within each family; (2) had BC_GF > 0.8; (3) MMAF < 0.01 and (4) CADD > 20 were selected for further analysis.

In two families, the WES-4 and WES-2 families Br2 and Br18, no variants were observed after applying the criteria. In the remaining 20 families, 544 variants in 521 genes were observed (Tables 2, S1–S2), where the majority of the variants were missense (n = 506, Table S2). There were a total of 38 variants with potential pathogenic effect (stop-gain, splicing and frameshift indels), where 20 variants were detected in four of the 12 WES-2s families (Br10, Br12, Br21 and Br22) (Table S1).

**Table 2:** Overview of risk variants with MMAF < 0.01, CADD > 20 and shared by all family members.

	Unique variants	Total calls	WES-4s	WES-3s	WES-2s	WES-1s
Number of families	20	20	2	5	12	1
Stopgain	22	22	1	0	15	6
Splicing	3	3	0	0	3	0
Frameshift deletion	9	9	0	3	6	0
Frameshift insertion	4	4	0	0	3	1
All missense	506	508	12	106	280	110
Total	544	546	13	109	307	117

WES-4s: families with 4 sequenced individuals; WES-3s: families with 3 sequenced individuals; WES-2s: families with 2 sequenced individuals; WES-1s: families with 1 sequenced individual.

Most of the deleterious variants were stop-gain (n = 22) and mainly detected in WES-2s families (n = 15). Two of the stop-gain variants were detected in genes that are involved in DNA damage response, (1) rs146594026 (c.C2152T, p.(Q718*)) in the *EXO1* gene that was detected in the WES-1 family Br24 and (2) rs147021911 (c.C5101T, p.(Q1701*)) in the *FANCM* gene that was detected in the WES-2 family Br21 (Table S1). Furthermore, 29 missense variants with CADD > 30 were detected in the BC families (Table S2). Among those 29 were rs28363218 (c.C604T, p.(R202C)) in the *RAD54L* gene detected in the WES-2 family Br22, chr2:216240089A > G (c.T5462C, p.(F1821S)) in the *FN1* gene detected in the WES-2 family Br12, rs544274181 (c.G1382A, p.(R461H)) in the *MET* gene detected in the WES-1 family Br24 and rs138942541 (c.G331T, p.(D111Y)) in the *ECD* gene detected in the WES-2 family Br23 (Table S2).

Recurrent genes, defined as genes segregating variants in more than one family, were seen among the BC families where we identified 46 variants located in 23 genes (Table S3). Two of the variants were detected in two families each: (1) rs142493383, in *ALPP* gene in families Br11 and Br24, and (2) rs200175537 in the *CLEC16A* gene in families Br12 and Br17. The *DNAH14* and *OBSCN* genes harbored three missense variants each, while we observed two variants in the remaining 19 genes. All variants were missense, apart from one stop-gain variant seen in the *LHCGR* gene in the WES-2 family Br19, and two frameshift deletions in the *RTN3* and *TTLL12* genes seen in the WES-2 families Br 22 and Br10, respectively (Table S3).

To further identify the most likely high-risk variants, a stricter criterion was applied to identify very rare and high impact variants in the larger families with sequencing data from 3 to 4 family members. The variants with MMAF < 0.001 and CADD > 25 were considered the most likely high-risk variants. In total, 22 variants in 22 genes were identified in six of the nine WES-3s and WES-4s families (Table 3). All variants except one were detected in the WES-3 families, and all but two were missense. Most high-risk mutations were detected in family Br7, followed by families Br9, Br5 and Br6 (n = 6, 5, 4 and 4, respectively) (Table 3). A stop-gain variant, rs143701013 (c.C889T, p.(R297*)), in the last exon of the *ZNF563* gene was observed in the WES-4 family Br3 where one individual was a homozygote carrier, and a frameshift deletion, rs769623079 (c.631_632del, p.(C211fs)), was seen in exon 7 in the *FANK1* gene in the WES-3 family Br5 (Tables 3, S1).

**Table 3:** High-risk variants with MMAF < 0.001, CADD > 25 and shared by all family members within families of 3 and 4 sequenced individuals.

Gene	Location Ref > Alt SNP-id	Function	Change	MMAF	CADD	WES	Family
*ZNF563*	chr19:12429950G > A rs143701013	Stopgain	NM_145276:exon4 c.C889T:p.R297X	3.0E−04	33	WES-4s	Br3
*PTPRF*	chr1:44086600T > C na	Missense	NM_130440:exon31 c.T5429C:p.I1810T	0	27.9	WES-3s	Br5
*PIWIL2*	chr8:22161596G > A na	Missense	NM_001135721:exon11 c.G1244A:p.G415D	0	29	WES-3s	Br5
*FANK1*	chr10:127693544TG >- rs769623079	Deletion	NM_145235:exon7 c.631_632del:p.C211fs	1.5E−05	na	WES-3s	Br5
*SBNO2*	chr19:1112229G > A rs745886953	Missense	NM_001100122:exon19 c.C2416T:p.R806C	3.0E−04	28.6	WES-3s	Br5
*ZNF862*	chr7:149557832G > A na	Missense	NM_001099220:exon7 c.G1583A:p.C528Y	0	25.7	WES-3s	Br6
*ASIC3*	chr7:150746421A > G rs201385813	Missense	NM_004769:exon1 c.A449G:p.Y150C	2.0E−04	25.2	WES-3s	Br6
*BMPER*	chr7:34192769A > C rs758133020	Missense	NM_133468:exon16 c.A1942C:p.N648H	5.0E−04	25.9	WES-3s	Br6
*HYPK*	chr15:44093970A > G rs200501830	Missense	NM_016400:exon4 c.A356G:p.N119S	4.0E−04	25.8	WES-3s	Br6
*EMX1*	chr2:73161012C > A rs766243607	Missense	NM_004097:exon3 c.C802A:p.H268N	1.0E−04	26.7	WES-3s	Br7
*EPPK1*	chr8:144942386C > T rs201157982	Missense	NM_031308:exon2 c.G5036A:p.R1679H	5.0E−04	31	WES-3s	Br7
*KIF26A*	chr14:104618716C > T rs759188299	Missense	NM_015656:exon3 c.C653T:p.T218M	5.0E−04	25.7	WES-3s	Br7
*PRR14*	chr16:30666219C > T rs576330025	Missense	NM_024031:exon8 c.C928T:p.R310C	9.0E−04	29.9	WES-3s	Br7
*PHLDB3*	chr19:44005948G > T rs773676224	Missense	NM_198850:exon4 c.C472A:p.L158M	5.0E−04	25.8	WES-3s	Br7
*LIG1*	chr19:48643245G > C rs760308186	Missense	NM_001289064:exon10 c.C866G:p.A289G	5.0E−04	28.3	WES-3s	Br7
*ANKRA2*	chr5:72850162C > T na	Missense	NM_023039:exon7 c.G790A:p.V264I	0	28.5	WES-3s	Br8
*ARAP1*	chr11:72415241G > A rs770843799	Missense	NM_015242:exon12 c.C1213T:p.R405C	6.7E−05	28.4	WES-3s	Br8
*EOMES*	chr3:27761701T > G rs745642069	Missense	NM_001278182:exon2 c.A997C:p.K333Q	3.0E−04	26.3	WES-3s	Br9
*DAAM2*	chr6:39869622C > T rs553394639	Missense	NM_001201427:exon25 c.C3016T:p.R1006W	4.0E−04	28.6	WES-3s	Br9
*YLPM1*	chr14:75248152A > T rs45550132	Missense	NM_019589:exon4 c.A1406T:p.Y469F	2.0E−04	26	WES-3s	Br9
*GALC*	chr14:88434679G > A rs756352952	Missense	NM_001201401:exon7 c.C839T:p.S280F	1.0E−04	33	WES-3s	Br9
*GIPC3*	chr19:3586860G > T rs775765891	Missense	NM_133261:exon3 c.G460T:p.G154C	7.7E−05	28.8	WES-3s	Br9

Location according to hg19, SNPid according to dbSNP150, Change shows transcript, exon, amino acid change and protein change. MMAF: Max minor allele frequency indicates the highest minor allele frequency in 20 population (see methods). WES-4s: families with 4 sequenced individuals; WES-3s: families with 3 sequenced individuals.

### FANCM stop-gain variant was observed in BC patients from four BC families

Finally, we searched for rare variants with high CADD that were observed in several BC patients, although not segregating within the families. In total, 15 variants with MMAF < 0.01 and CADD > 25 and detected in at least three families were seen (Table S4). A stop-gain variant, rs147021911 (c.C5101T, p.(Q1701*)) in the *FANCM* gene, was found in 6 individuals from four families. The variant was detected in both family members of the WES-2 family Br21, as well as in two individuals from the WES-4 family Br3 and one individual from each of the two WES-2 families Br18 and Br23 (Table S4). Similarly, a missense variant, rs1065746 (c.G3244C, p.(D1082H)) in the *HTT* gene was observed in both family members of the WES-2 family Br20, as well as in two individuals from the WES-4 family Br3 and two individuals from the WES-3 family Br8 (Table S4). A missense variant, rs149133270 (c.G1379A, p.(R460Q)), in the *MPO* gene found in the WES-1 Br24, was also seen in four individuals from two WES-4 families and one WES-3 family. The remaining seven variants were seen in three families each (Table S4).

## Discussion

To identify known and novel causative variants that could contribute to hereditary BC, we exome sequenced a selection of patients from 24 Swedish BC families. First, we screened for variants with a pathogenic or possible pathogenic consequence in known BC predisposing genes. Secondly, we searched for rare variants that segregated in BC families with predicted high impact and which could have contributed to the disease.

Three pathogenic variants in the *BRIP1*, *PALB2* and *CHEK2* genes were found in five families. Since loss-of-function variants in the *BRIP1* and *PALB2* genes increase the risk of BC and OC^28,29^, these variants were considered to be the main cause of the increased cancer risk in these two families. The c.1100delC variant in the *CHEK2* gene is a well-known variant considered to confer an increased risk of BC^30^. However, the risk is considered moderate, and it cannot be concluded that this variant solely explains the BC risk in these families. Several variants with uncertain significance were detected in the BC families. However, further analyses are needed to determine their contribution to BC.

To identify new BC predisposing genes, strict filtering was performed on the remaining families. Variants shared by all family members and with deleterious effects or high CADD were as critera for possible high-risk predisposing variants in the families. In total, 38 deleterious variants and over 500 missense variants were seen in the families, most of them in WES-2s families. Of the 506 missense variants, 29 had CADD > 30 and were considered strong candidates to predispose to the disease.

We observed variants located within genes that have previously been linked to BC. The *FANCM* gene is part of the Fanconi anemia complementation group, which includes the well-known BC risk genes *BRCA2*, *BRIP1* and *PALB2*. Like those genes, *FANCM* is involved in DNA double-strand break repair and has been linked to BC^31–33^. The stop-gain variant in the *FANCM* gene seen here in Swedish BC patients has previously been reported in BC patients^31,32^ including familial cases^31^, and is has been sugressed to be common in Finnish triple-negative BC patients^32^. Here, it was found in four families, although not in all family members, suggesting this variant can be a risk factor for BC.

Several other interesting variants were seen in genes that could contribute to BC, such as the *RAD54L* and *FN1* genes. The *RAD54L* gene is involved in DNA recombination, along with the *RAD51C* and *RAD51D* genes, and has been linked to BC^34^. The variant is located in exon 7 that contains helicase motif I and Ia^35,36^. These motifs identify helicases and are important for protein function. The *FN1* gene is involved in cell adhesion, the oncogene *MET*, and the *ECD* gene, a cell cycle regulatior, are all interesting candidates and have previvously been reported in BC^37–41^. Further studies are needed to understand their contribution to BC.

This study has several limitations that need to be considered. The cohort consists of a limited number of BC patients and families that were exome sequenced. Therefore, variants outside of the exons are not analyzed here, and our analysis is limited to single nucleotide variants and smaller indels. Furthermore, a strict selection criterion was applied to identify novel risk genes that are rare and assumed with a high impact, thereby excluding more common variants that might contribute to the disease. Since part of our criteria was that variants needed to segregate within all family members sequenced, we have a bias towards more variants detected in smaller families and families containing close relatives. Finally, only affected family members were analyzed. Including unaffected family members could have been beneficial regarding variant filtering.

## Conclusions

Identifying new risk genes is important for genetic counseling of BC families and to determine the cancer risk in family members. Here, we analyzed pathogenic variants in known and novel BC predisposing genes in families with a strong history of BC. Several interesting candidate genes were observed that could have contributed to the disease in these families. Further studies are needed to evaluate the contribution of those genes and variants to and increased BC risk.

## Supplementary Information

Supplementary Tables.

## Data Availability

Access to the data is controlled. Variants that fulfilled our selection criteria can be found in the supplementary tables. However, Swedish laws and regulations prohibit the release of individual and personally identifying data. Therefore, the whole data cannot be made publicly available. The data that support the findings of this study are available from the corresponding authors upon a reasonable request.

## Abbreviations

BC: Breast cancer
BC_GF: Breast cancer genotype frequency
GWAS: Genome-wide association studies
MMAF: Maximum minor allele frequency
MMR: Mismatch repair
WES: Whole-exome sequencing
WGS: Whole-genome sequencing
